# Supplementary material for: Modeling glioblastoma heterogeneity as a dynamic network of cell states
Source: Mol Syst Biol. 2021 Sep 16;17(9):e10105. doi: 10.15252/msb.202010105 (PMC8444284; doi:10.15252/msb.202010105)
Supplement: Supplementary file 6 — Source Data for Figure 5 [file MSB-17-e10105-s004.zip › Figure5A_sourcedata/GSEA_3017/nosigdb_stateB.GseaPreranked.1622795021725/RICHARDS_CAHOY_JNEUROSCI_2015_ASTRO_IN_VIVO.html]

Details for gene set RICHARDS\_CAHOY\_JNEUROSCI\_2015\_ASTRO\_IN\_VIVO[GSEA]

|  || Dataset | state43017 |
| Phenotype | NoPhenotypeAvailable |
| Upregulated in class | na\_neg |
| GeneSet | RICHARDS\_CAHOY\_JNEUROSCI\_2015\_ASTRO\_IN\_VIVO |
| Enrichment Score (ES) | -0.19330992 |
| Normalized Enrichment Score (NES) | -1.9813869 |
| Nominal p-value | 0.0 |
| FDR q-value | 0.015053624 |
| FWER p-Value | 0.048 |
Table: GSEA Results Summary

  

Fig 1: Enrichment plot: RICHARDS\_CAHOY\_JNEUROSCI\_2015\_ASTRO\_IN\_VIVO      
 Profile of the Running ES Score & Positions of GeneSet Members on the Rank Ordered List

  

| PROBE | GENE SYMBOL | GENE\_TITLE | RANK IN GENE LIST | RANK METRIC SCORE | RUNNING ES | CORE ENRICHMENT || 1 | UPP1 |  |  | 74 | 0.572 | -0.0896 | No |
| 2 | CYR61 |  |  | 145 | 0.465 | -0.1770 | Yes |
| 3 | LYPD1 |  |  | 151 | 0.459 | -0.1684 | Yes |
| 4 | GPC6 |  |  | 159 | 0.454 | -0.1628 | Yes |
| 5 | PDE1C |  |  | 166 | 0.445 | -0.1562 | Yes |
| 6 | NCAM1 |  |  | 178 | 0.436 | -0.1572 | Yes |
| 7 | ELN |  |  | 182 | 0.433 | -0.1465 | Yes |
| 8 | CKAP5 |  |  | 192 | 0.426 | -0.1449 | Yes |
| 9 | CADM1 |  |  | 201 | 0.414 | -0.1423 | Yes |
| 10 | CHST2 |  |  | 224 | 0.397 | -0.1609 | Yes |
| 11 | SLC1A3 |  |  | 237 | 0.388 | -0.1651 | Yes |
| 12 | PPP1CB |  |  | 246 | 0.385 | -0.1635 | Yes |
| 13 | EPS8 |  |  | 257 | 0.371 | -0.1653 | Yes |
| 14 | RAD23A |  |  | 258 | 0.371 | -0.1523 | Yes |
| 15 | ASRGL1 |  |  | 266 | 0.366 | -0.1499 | Yes |
| 16 | ZFP36L2 |  |  | 279 | 0.358 | -0.1551 | Yes |
| 17 | RAMP1 |  |  | 285 | 0.357 | -0.1500 | Yes |
| 18 | PRKDC |  |  | 306 | 0.344 | -0.1676 | Yes |
| 19 | ABHD3 |  |  | 310 | 0.343 | -0.1601 | Yes |
| 20 | PRRX1 |  |  | 318 | 0.339 | -0.1585 | Yes |
| 21 | PRDX6 |  |  | 323 | 0.338 | -0.1526 | Yes |
| 22 | EMP2 |  |  | 333 | 0.333 | -0.1543 | Yes |
| 23 | MACF1 |  |  | 342 | 0.328 | -0.1547 | Yes |
| 24 | PHGDH |  |  | 344 | 0.328 | -0.1447 | Yes |
| 25 | EIF5 |  |  | 346 | 0.327 | -0.1347 | Yes |
| 26 | NRGN |  |  | 348 | 0.326 | -0.1248 | Yes |
| 27 | PNRC2 |  |  | 364 | 0.321 | -0.1358 | Yes |
| 28 | MID1 |  |  | 375 | 0.317 | -0.1395 | Yes |
| 29 | TGFB2 |  |  | 388 | 0.312 | -0.1463 | Yes |
| 30 | TTYH3 |  |  | 396 | 0.309 | -0.1459 | Yes |
| 31 | TMEM132A |  |  | 406 | 0.306 | -0.1485 | Yes |
| 32 | PPP1R14C |  |  | 407 | 0.305 | -0.1378 | Yes |
| 33 | PPP3CA |  |  | 417 | 0.300 | -0.1406 | Yes |
| 34 | CD47 |  |  | 419 | 0.300 | -0.1316 | Yes |
| 35 | GAMT |  |  | 434 | 0.295 | -0.1420 | Yes |
| 36 | PPP1R9A |  |  | 435 | 0.295 | -0.1317 | Yes |
| 37 | CHPT1 |  |  | 445 | 0.294 | -0.1347 | Yes |
| 38 | SREBF1 |  |  | 453 | 0.292 | -0.1349 | Yes |
| 39 | CEP350 |  |  | 455 | 0.291 | -0.1261 | Yes |
| 40 | SYNE1 |  |  | 458 | 0.291 | -0.1189 | Yes |
| 41 | TTYH1 |  |  | 469 | 0.287 | -0.1237 | Yes |
| 42 | NR2F1 |  |  | 473 | 0.287 | -0.1181 | Yes |
| 43 | APLP1 |  |  | 480 | 0.285 | -0.1170 | Yes |
| 44 | APOLD1 |  |  | 487 | 0.284 | -0.1159 | Yes |
| 45 | CTDSPL2 |  |  | 495 | 0.282 | -0.1164 | Yes |
| 46 | CAMK2N1 |  |  | 505 | 0.280 | -0.1199 | Yes |
| 47 | CTDSPL |  |  | 540 | 0.274 | -0.1607 | Yes |
| 48 | POLR2A |  |  | 545 | 0.273 | -0.1571 | Yes |
| 49 | DKK3 |  |  | 549 | 0.273 | -0.1520 | Yes |
| 50 | CALU |  |  | 552 | 0.272 | -0.1454 | Yes |
| 51 | RORB |  |  | 555 | 0.271 | -0.1389 | Yes |
| 52 | BRD8 |  |  | 562 | 0.270 | -0.1383 | Yes |
| 53 | GGH |  |  | 564 | 0.270 | -0.1303 | Yes |
| 54 | PBX1 |  |  | 567 | 0.270 | -0.1238 | Yes |
| 55 | PCDH19 |  |  | 581 | 0.268 | -0.1337 | Yes |
| 56 | CCDC18 |  |  | 586 | 0.267 | -0.1303 | Yes |
| 57 | GNG12 |  |  | 587 | 0.267 | -0.1209 | Yes |
| 58 | ST8SIA5 |  |  | 588 | 0.266 | -0.1116 | Yes |
| 59 | IFRD1 |  |  | 594 | 0.264 | -0.1098 | Yes |
| 60 | IDH2 |  |  | 598 | 0.263 | -0.1050 | Yes |
| 61 | OXCT1 |  |  | 614 | 0.261 | -0.1181 | Yes |
| 62 | NDE1 |  |  | 623 | 0.259 | -0.1209 | Yes |
| 63 | CHCHD3 |  |  | 628 | 0.259 | -0.1178 | Yes |
| 64 | SCCPDH |  |  | 638 | 0.257 | -0.1221 | Yes |
| 65 | CHN1 |  |  | 645 | 0.256 | -0.1220 | Yes |
| 66 | MAGI3 |  |  | 648 | 0.256 | -0.1160 | Yes |
| 67 | VAV3 |  |  | 650 | 0.256 | -0.1085 | Yes |
| 68 | PIGX |  |  | 657 | 0.255 | -0.1085 | Yes |
| 69 | PCBP2 |  |  | 660 | 0.255 | -0.1025 | Yes |
| 70 | RPN1 |  |  | 662 | 0.254 | -0.0951 | Yes |
| 71 | PXMP2 |  |  | 663 | 0.253 | -0.0863 | Yes |
| 72 | MCC |  |  | 664 | 0.253 | -0.0774 | Yes |
| 73 | LONRF2 |  |  | 677 | 0.251 | -0.0864 | Yes |
| 74 | HMGCR |  |  | 683 | -0.257 | -0.0848 | Yes |
| 75 | CHD7 |  |  | 706 | -0.284 | -0.1074 | Yes |
| 76 | ID4 |  |  | 708 | -0.285 | -0.0989 | Yes |
| 77 | PCDH10 |  |  | 713 | -0.305 | -0.0942 | Yes |
| 78 | MARCKSL1 |  |  | 716 | -0.311 | -0.0862 | Yes |
| 79 | ZEB1 |  |  | 717 | -0.317 | -0.0751 | Yes |
| 80 | MXD4 |  |  | 731 | -0.384 | -0.0809 | Yes |
| 81 | IDI1 |  |  | 734 | -0.397 | -0.0700 | Yes |
| 82 | SQLE |  |  | 735 | -0.405 | -0.0558 | Yes |
| 83 | HMGCS1 |  |  | 739 | -0.418 | -0.0456 | Yes |
| 84 | BTG1 |  |  | 741 | -0.423 | -0.0323 | Yes |
| 85 | FABP5 |  |  | 759 | -1.641 | -0.0000 | Yes |
Table: GSEA details [plain text format]

  

Fig 2: RICHARDS\_CAHOY\_JNEUROSCI\_2015\_ASTRO\_IN\_VIVO: Random ES distribution      
 Gene set null distribution of ES for **RICHARDS\_CAHOY\_JNEUROSCI\_2015\_ASTRO\_IN\_VIVO**
